# Bayesian statistical modelling of human protein interaction network incorporating protein disorder information

**DOI:** 10.1186/1471-2105-11-46

**Published:** 2010-01-25

**Authors:** Svetlana Bulashevska, Alla Bulashevska, Roland Eils

**Affiliations:** 1 Theoretical Bioinformatics Department, German Cancer Research Center (DKFZ), Im Neuenheimer Feld 280, 69120 Heidelberg, Germany; 2 Department of Bioinformatics and Functional Genomics, Institute of Pharmacy and Molecular Biotechnology (IPMB) and Bioquant, University of Heidelberg, Germany

## Abstract

**Background:**

We present a statistical method of analysis of biological networks based on the exponential random graph model, namely p2-model, as opposed to previous descriptive approaches. The model is capable to capture generic and structural properties of a network as emergent from local interdependencies and uses a limited number of parameters. Here, we consider one global parameter capturing the density of edges in the network, and local parameters representing each node's contribution to the formation of edges in the network. The modelling suggests a novel definition of important nodes in the network, namely *social*, as revealed based on the local *sociality *parameters of the model. Moreover, the sociality parameters help to reveal organizational principles of the network. An inherent advantage of our approach is the possibility of hypotheses testing: *a priori *knowledge about biological properties of the nodes can be incorporated into the statistical model to investigate its influence on the structure of the network.

**Results:**

We applied the statistical modelling to the human protein interaction network obtained with Y2H experiments. Bayesian approach for the estimation of the parameters was employed. We deduced *social *proteins, essential for the formation of the network, while incorporating into the model information on protein disorder. *Intrinsically disordered *are proteins which lack a well-defined three-dimensional structure under physiological conditions. We predicted the fold group (ordered or disordered) of proteins in the network from their primary sequences. The network analysis indicated that protein disorder has a positive effect on the connectivity of proteins in the network, but do not fully explains the interactivity.

**Conclusions:**

The approach opens a perspective to study effects of biological properties of individual entities on the structure of biological networks.

## Background

The advent of high-throughput technologies allows the large-scale identification of cellular components and their interactions. This wealth of experimental data is assembled in biological networks (transcriptional regulatory, protein-protein interaction, metabolic, signaling, phosphorylation networks etc.). The question of systems biology araises: do the structural, architectural principles of biological networks can reveal functional properties of cellular systems. Analysis of biological networks has become one of the emergent topics. It was suggested that biological networks are *scale-free *with the majority of nodes having a small number of connections but with relatively fewer *hubs *possessing a high degree of connectivity [1,2]. Starting from the work of Jeong *et al *[3], which stated that highly connected proteins are more likely to be lethal when knocked out, many works showed that hubs represent vulnerable points in the cell. Previous approaches, however, used different, rather subjective definitions of hubs. Some of them defined hubs as the top 20% of high-degree nodes [4], while other defined them as those interacting with ≥ 10 partners [5].

Beyond a purely local notion of centrality such as the node degree, more global centrality measures incorporating network-wide properties have attracted much interest. E.g. *betweenness centrality *which measures the total number of shortest pathes going through a node [6,7]. Further centrality measures like *eccentricity, centroid *etc. are described in [8,9]. Recently, the *pairwise diconnectivity index *of a network's element was proposed, defined as the fraction of initially connected pairs of nodes which become disconnected if the element is removed from the network [10]. The new measure quantifies the importance of an individual element in sustaining communication in the network. Hence, different measures result in different scoring of nodes in a network and lead to different hypotheses about the importance of biological entities.

Previous approaches for the analysis of biological networks were descriptive, graph-based ones. In this paper, a statistical approach is employed, originating from social network analysis field [11]. It is important to recognize that biological processes underlying the network data as well as experimental measurements are of stochastic nature: some of the interactions might be unreliable and some of the real relations might be missed or not measured. This concern leads to the employment of the statistical approach based on probability distributions. A probabilistic model is defined which tries to predict edge probabilities and captures network properties summarizing them in form of statistical parameters. By learning the model from available network data, estimates of the parameters are obtained. I.e. the empirical network is treated as an outcome of a statistical process that generated the network data. Thus, generic questions can be addressed: How the biological network emerged? Are there local biological processes that generated the network? In the statistical approach, the global network structure emerges as an agglomeration of local structural configurations, which rely on local interdependencies of edges. In fact, it is the dependency that may explain the deviation of real biological networks from random ones.

At the heart of the statistical modelling of networks is a general family of graph probability distributions called *exponential random graph models *(ERGMs) or the p*-model [12]. Each possible edge in a network is represented by a probabilistic variable and the network model expresses the value of each variable as a stochastic function of network structural properties. Various dependencies among the network variables are considered reflecting the interactive nature of processes from which a given network emerges. The dependencies give rise to different local structural configurations and correspond to different classes of models. The simplest dependency structure assumes no dependencies among the network variables (all edges are independent). Allowing the probabilities of edges to be equil implies the class of *Bernoulli graphs *or *Erdős-Rényi graphs *(p0-model). The structural configuration considered in this model is just a single edge. Further on, the p1-model (*dyadic independence *model) assumes the dependency between reciprocated edges connecting two nodes in a directed graph [13]. The *Markov random graph *model considers Markov dependencies, whereby two possible edges are assumed to be dependent if they share a node. (Such type of dependency resembles a Markov spatial process). The assumption leads to the consideration in the network model of three-nodal network structures like *stars *(2-path, 2-star, 3-star etc.) and *triangles*. The Hammersley-Clifford theorem [14] yields the expression of the graph probability distribution in terms of *network statistics*, being counts of local network configurations. In the formulation of the model, each statistic comes with its own parameter reflecting the importance of the corresponding configuration in the network under study. They are a kind of 'regression coefficients' which, when positive, indicate that a particular network configuration is relevant for the emergence of edges in the network. The choice of statistics present in the model embodies different assumptions about relevant local processes that might generate the network. Recently, new statistics were proposed including higher-order triangulations, *alternating k-stars, geometrically weighted degree *(GWD) [15,16].

Exponential random graph models for biological networks were first explored in [17]. There, several networks (RegulonDB *E.coli*, ChIP-chip *S.cerevisiae *etc.) were fitted using 2-5-degree statistics, 2-star, edges and GWD, while iteratively increasing the complexity of the models. The results show that only basic statistics, namely edges and 2-star, have a positive effect, determining the network.

In this paper, we leave apart complex structural configurations and, instead, turn to a node-oriented model, namely to *p2-model*, which is a generalization of the *p1-model *[18]. The p1-model considers *dyads *(a pair of directed edges between two nodes) and represents the probability of any dyad as the function of global features of the graph (density and tendency towards reciprocity) and of individual nodal features (tendencies to send edges and to receive them). The model specification thus includes two global parameters - *density *(also called *edges*) and *reciprocity*, as well as local, node-specific parameters: *expansiveness *and *attractiveness*. The density determines the probability of an edge between a pair of nodes irrespective of individual characteristics of the nodes and captures the overall formation of edges in the network. The attractiveness and expansiveness describe the abilities of a node to attract and to produce edges beyond the overall density, hence individual nodal contributions to the network emergence. Estimates of the expansiveness and attractiveness parameters can be used to rank the nodes and to reveal the most important nodes in the network from the perspective of the statistical modelling.

Given the node-specific parameters, the p1-model implies the conditional independence between dyads. The p2-model is an extension of the p1-model in that the nodal parameters are modelled as *random effects*, a formulation that makes it possible to include into the model node- and dyad-specific covariates, as will be shown below.

Although the p1-model was defined for directed graphs, it can be modified to be applicable for undirected graphs. Then, the local, node-specific parameters are called *sociality *and reflect the propensity of an individual node to be connected to other nodes in the network. We term *social *nodes those with positive sociality parameters i.e. positively influencing the formation of edges in the network.

In this paper, we apply the undirected graph p2-model to a human protein interaction network. We assess the node-specific parameters of the model and use them to infer *social *nodes, which are important for the emergence of the network. Thus, our view of essential nodes in biological networks is based not on the descriptive measures, but, alternatively, on the parameters of the statistical model learned from network data.

An apparent advantage of the present approach is the possibility to introduce exogeneous biological knowledge into the network model and to study it in the light of structural properties of the network. This permits to investigate important biological hypotheses and to examine, what biological properties have effect on the connectivity of proteins in the network. In this paper, we introduce into the protein interaction network model the information on protein disorder and study, if this property influences the protein interactivity. Further benefit of the node-specific sociality parameters is that they provide a basis for partitioning the nodes into groups of structural similarity. One important question is: How many such structural groups are contained in the network? The connectivity pattern between the groups displays organizational properties of the network, in abstraction from the level of individual proteins. We analyze the protein interaction network to reveal its organizational principles.

It was well established in biology that proteins fold to their unique native conformations as determined by their amino acid sequences, and that each protein's function originates from a specific three-dimentional (3D) strucuture. In the last decade, however, numerous biologicaly active proteins were found that fail to maintain stable ordered 3D-structures under physiological conditions [19-22]. These proteins are called natively unfolded or *intrinsically disordered*. Bioinformatics analysis has revealed that about 25-30% of eukaryotic proteins are mostly disordered, and that more than half of eukaryotic proteins have long regions of disorder [23,24]. It was predicted that 70% of signaling proteins and vast majority of cancer- and cardiovascular disease-associated proteins have long disordered regions [25,26]. It was shown that disordered proteins play a number of crucial roles in regulation, signaling and control processes, many post-translational modifications (ubiquitination, methylation, phosphorylation etc.) occur within the regions of intrinsic disorder [27]. In [28-30], the authors revealed 238 Swiss-Prot functional keywords positively correlated with long disordered regions in proteins. The common view now is that disordered regions carry out a large variety of functions [31,32].

It is postulated that the intrinsic disorder plays an important role in the interactivity of proteins [33]. The intrinsically disordered proteins can bind to large numbers of diverse targets or can facilitate other proteins binding to many targets. Haynes *et al *demonstrate that intrinsic disorder is a distinctive and common characteristic of eukaryotic hub proteins [5]. Dosztanyi *et al *[34] and Patil and Nakamura [35] investigate the structural properties of hubs that enable them to interact with multiple proteins and conclude that global flexibility and extended interaction surface provided by disordered regions play a significant role in the binding ability of hubs. In this paper, we study the effect of disorder on the connectivity of proteins.

## Methods

### Data

#### The human protein interaction network

We used the data of Stelzl *et al *generated by automated yeast two-hybrid (Y2H) interaction mating [36]. From the totally identified 3186 interactions among 1705 proteins, the authors selected 911 interactions among 401 proteins for the high-confidence protein interaction network. They developed a scoring system with 6 criteria including the existence of orthologous protein interactions in *D. melanogaster*, *C. elegans *and *S. cerevisiae*, the proteins shareness of GO annotations etc. Only the interactions which collected 3 or more points by the evaluation with 6 criteria were selected. We use this high-scoring protein interaction network for our analysis. Additional File 1 contains the list of the 401 underlying proteins.

#### Protein sequences

The primary sequences of the proteins used for the prediction of them into disordered/ordered were downloaded from NCBI [37].

### Prediction of protein disorder

To predict the fold group (ordered or disordered) of the proteins we used POODLE, freely available at [38]. POODLE is a set of programs for predicting protein disorder from amino acid sequences using machine learning approaches. POODLE provides three types of disorder prediction according to the length of the target disorder. POODLE-S version puts emphasis on predicting short disorder regions [39]. POODLE-L version focuses on predicting long disorder regions, mainly longer than 40 consecutive amino acids [40]. POODLE-W version predicts if a protein is mostly disordered [41]. We used POODLE-W to classify our 401 proteins into disordered or ordered. Additional File 2 contains the disorder probability outputed by POODLE-W for each protein. Based on these probabilities, using the threshold 0.7, we obtained 130 disordered proteins (see the list of these proteins in Additional File 3).

### Bayesian statistical analysis of the network

#### Network model

In the statistical modelling approach, the network with *n *nodes is represented by a random variable **X**, a realization of **X **is denoted by **X **= **x**. Let binary outcome *X*_*ij *_= 1 if node *i *has a directed edge to node *j*, and *X*_*ij *_= 0 otherwise, then **x **is a binary data matrix (*X*_*ij*_). The exponential random graph model has the following general form:

where *z*_*p*_(**x**) is the network statistic of type *p*, ***θ***_*p *_is the parameter associated with *z*_*p*_(**x**) and *κ*(***θ***) is a normalizing constant that ensures that the probabilities sum to unity [42]. The parameters ***θ ***are the unknown 'regression coefficients' which need to be estimated.

It is the normalizing constant, which makes the maximum likelihood estimation of the random graph models intractable, since it is defined over the entire graph space with 2^*n*(*n*-1) ^possible directed graphs. Thus, for the estimation of p*-models, alternative model formulations and approximate estimation techniques evolve. The *maximum pseudolikelihood *estimation approach (MPLE) uses an approximate likelihood function [43]. The MC-MLE applies Markov Chain Monte Carlo stochastic simulation technique for the maximum likelihood estimation [44].

In the present paper, we consider a special class of the ERGMs, the p2-model. The model allows to employ Bayesian model inference using Gibbs sampling as proposed in [45].

Firstly, we introduce the p1-model for directed graphs and then show how it can be modified for the undirected case. The p2-model is presented in the next section.

The p1-model considers the relationships between two nodes, namely there are three relations possible: no edges, an asymmetric edge, or a reciprocated edge. The p1-model seeks to predict the probability of each of these kinds of relations for each pair of nodes. The unit of analysis for the model is *dyad D*_*ij *_= (*X*_*ij*_, *X*_*ji*_), i.e. the pair of edges between two nodes *i *and *j*, for 1 ≤ *j *≤ *n*. Each dyad has four possible outcomes: (0, 0), (1, 0), (0, 1), (1, 1). Let

with *m*_*ij*_*+ a*_*ij*_*+ a*_*ji*_*+ n*_*ij *_= 1. Here, *m*_*ij *_stands for the probability of a reciprocated (mutual) relation, *a*_*ij *_for an assymetric relation and *n*_*ij *_for no relation between nodes *i *and *j*. The p1-model assumes that the dyads are mutually independent, then the joint probability for the data matrix (*X*_*ij*_) is written as:

By taking logarithms and exponentiating, one obtains

where

and

The *log-odds-ratio θ*_*ij *_divides the sample cases where there is an edge from *i *to *j *by cases where there is no edge and, therefore, measures the likelihood of an edge. The log-odds-ratio *ϕ*_*ij *_divides the symmetric cases by the assymetric cases and, intuitively, measures the tendency for reciprocation of the edge (*i, j*). To overcome the problem of too many parameters, it was postulated that

Henceforth, *ϕ *is the global parameter that measures the degree of reciprocity in the entire network; *θ*_*ij *_is splitted into the global and local node-specific effects. The global parameter *θ *(called *density*) measures the overall degree of forming edges in the population. Each local parameter *α*_*i *_represents the propensity of the node *i *to send edges (*expansiveness*). The local parameter *β*_*j *_represents the ability of the node *j *to attract edges (*attractiveness*). A positive value of *α*_*i *_or *β*_*i *_indicates that the corresponding node *i *is more expansive or attractive, respectively, than a typical member of the population i.e. it contributes to the probability of the edge beyond the overall density in the network.

With the transformation of parameters, the distribution is then written as:

In this form the distribution belongs to the exponential family with the following statistics: *M *- the number of mutual (reciprocated) edges, *E *- the total number of edges, and Δ_*in*_*(i) *and Δ_*out*_*(i) *- the in- and out-degrees of node *i*. The equation shows, that the coefficient *θ *refers to the effect of the global density of the network on the probability of edges between two nodes, *ϕ *refers to the effect of the overall amount of reciprocity in the network on the probability of a reciprocated edge, *α *and *β *refers to the effect of each node's out-degree and in-degree on the probability that the node will have connections to other nodes. An equivalent log-linear formulation of the p1-model was suggested by Fienberg and Wasserman [46]. In this formulation, a dyad (*X*_*ij*_, *X*_*ji*_) is represented by four Bernoulli variables *Y*_*ij*00_, *Y*_*ij*10_, *Y*_*ij*01 _*and Y*_*ij*11 _as follows:

The p1-model is then described with the four log-linear equations:

for *i *<*j*. The first and the second equations describe the probability that two nodes will be connected with an asymmetric relation. The third is designed to predict the probability of a reciprocated relation and the last describes the probability of a null relation between nodes. In this formulation, the *scaling parameters λ *_*ij*_*= log*(*n*_*ij*_) play a role of the 'residual': if an edge is not mutual or asymmetric, it must be non-present.

*λ*s are fixed according to the constraint Σ_*k*, *l *_*Y*_*ijkl *_= 1.

In case of an undirected graph, the previously described model simplifies as follows. Instead of four, only two Bernoulli variables *Y*_*ij*0_, *Y*_*ij*1 _are considered:

Since there is no directed edges, the reciprocity parameter *ϕ *equals 0. The expansiveness and attractiveness parameters reduce to the parameter of only one type called *sociality*, which reflects the propensity of a node to be connected in an undirected graph. We denote it with *α*. The model is then defined by the following two equations predicting the probability of an edge between nodes *i *and *j *to be present or absent in the graph:(1)

for *i *<*j*. *θ *is the global density parameter. Positive values of *α*_*i *_or *α*_*j *_increase the probability that nodes *i *and *j *will be connected. Henceforth, the p1-model seeks to examine how edges between pairs of nodes relate to particularly important attributes of each node and of the network as a whole, i.e. the model includes the structural features of the network explicitly.

#### Extension of the network model (p2-model)

The concepts density and reciprocity of a network, as well as node-specific attractiveness and expansiveness are defined endogeneously i.e. based on the relations within the network. However, they can be linked to some exogeneous concepts using nodal (as well as edge-specific) attributes. This states one of the important advantages of the statistical modeling of networks. The binary network data can be related to nodal attributes while taking into account the specific network structure.

Particularly, the network model can be extended by modeling sociality parameters ***α ***as a linear regression:

where **X**_**i **_is the vector of size *m *≥ 1 of the attributes (i.e. explanatory variables or covariates) of node *i*, ***γ ***is the vector of the corresponding coefficients, and **A **are the so called random effects [18]. This formulation expresses the simple idea that socialities depend on the nodal attributes. Naturally, the attributes do not explain all variation in sociality parameters as is represented by the terms **A**. The components of **A **are modelled as normally distributed random variables with expectation 0 and variance ). This variance can be interpreted as the variance of the *α*'s left after taking into account the effect of the covariates **X**.

In this paper, we consider only one covariate - the disorder property of each node in the network, thus studying the effect of disorder on the nodal sociality. Generally, multiple attributes can be considered in the extended model. Also, in a similar manner, density and reciprocity parameters can be related to edge-specific attributes.

The specification of *α*s described here turns the original p1-model with fixed effects parameters into the random-effects p2-model [18]. The original p1-model assumes that the dyads are independent, i.e. the probability of edges between the nodes *i *and *j *is not affected by the presence (or absence) of edges involving any other pair of nodes. Obviously, this assumption is very constraining. The generalization of the p1-model including node-specific random effects relaxes the assumption of dyadic independence. Conditionally on the random effects, the dyads are then assumed to be mutually independent.

#### Bayesian inference of the network model

We employ a fully Bayesian approach to network modelling. Different to the maximum likelihood estimation, the Bayesian approach addresses the problem of learning the model from data as calculating the posterior probability of a model given data. Suppose that the data *D *has been generated by a model *m*. If *p*(*m*) is the prior probability of model *m*, then the posterior model probability by Bayes rule is

The marginal likelihood is defined as

where *p*(*θ*_*m*_|*m*) is the prior distribution of model parameters *θ*_*m *_for model *m *. The calculation of the marginal likelihood for the model considered here is intractable. Hence, Markov Chain Monte Carlo (MCMC) stochastic simulation techniques must be employed to facilitate the Bayesian model inference. MCMC simulation generates samples from the joint posterior distribution *p*(*m, θ_m_*|*D*) allowing to estimate the posterior parameter probabilities. Here, the MCMC technique *Gibbs sampling *is being applied. Gibbs sampling reduces the problem of dealing simultaneously with a large number of unknown parameters in a joint distribution into a simpler problem of dealing with one variable at a time, iteratively sampling each from its full conditional distribution given the current values of all other variables in the model. Bayesian approach for learning the network model using Gibbs sampling was described previously in [45].

We utilize the Linux version of the software OpenBUGS (BUGS stands for Bayesian Updating with Gibbs Sampling), the general purpose software for Gibbs sampling on graphical models [47]. OpenBUGS provides a declarative language for specifying a graphical model. The output of Markov chain simulation is used to summarize the posterior distribution of the variables of interest.

The Bayesian approach requires the specification of the model likelihood and of the prior probability distributions for the model parameters. Equations (1) define the *model likelihood*, where *Y*_*k *_are the data matrices to be calculated previously based on the observed data *X*. In a Bayesian approach, we can specify the prior distribution for the model parameters *hierarchically*, i.e. dependent on the *hyperparameters*. We define the prior probability distribution for the density parameter *θ *as a normal distribution with mean 0 and standard deviation *σ_θ _*(the operator ~ stands for 'is distributed as'):

Now we need to specify the prior distribution for the hyperparameter *σ_θ_*. Note, that in OpenBUGS, the normal distribution is parameterized by its *precision τ*, rather than its standard deviation *σ*, which are connected by *τ *= *σ*^-2^. We prescribe a gamma distribution for the precision parameter, that is a good practice, since gamma distribution is a conjugate prior distribution for the precision of the normal distribution. The specification is then written as:

To make the prior for *θ noninformative*, its standard deviation should be large, thus expressing large uncertainty. This is achieved by setting the hyperparameters in the gamma distribution as *a*_0 _= 0.001 and *b*_0 _= 0.001.

Within the statistical approach for network analysis proposed here, it is possible to include a node-level information into the network model and study the effects of this information on model parameters. We incorporate into the model the information on protein disorder for each protein in the network. The node-specific sociality parameters *α*_*i*_, *i *= 1,..., *n *are then defined as follows:

where *g*_*i *_is the binary variable, *g*_*i *_= 1 if the node *i *belongs to the group of disordered proteins and *g*_*i *_= 0 otherwise; *γ *represents the effect of the disorderdness on the sociality parameter and *a*_*i *_is the random effect. Note that although the attribute disorder is represented here with the binary valued variable, continuous or ordinal variables can be readily inserted as covariates into the model.

The parameter *γ *is modeled as follows:

The prior specification for the parameters *a*_*i *_is:

Again, the hyperparameters *a*_0 _and *b*_0 _are equal 0.001.

Note that conditionally on , , the parameters *θ *and *α*_*i *_are independent.

#### Monitoring convergence of the Markov chain

For summarizing and monitoring convergence of the Markov chain, the R-package CODA was used [48]. The Markov chain must be monitored for diagnosing a lack of convergence. We used Geweke's, Raftery and Lewis's, Gelman and Rubin's diagnostics. As proposed by Gelman and Rubin, a number of parallel runs of Markov chains should be carried out from different starting points. Convergence is diagnosed when the output from different Markov chains is indistinguishable. For parallel runs of Markov chains we used different initial values of the parameters.

#### Checking goodness-of-fit of the network model

The goodness-of-fit checking of the network model requires the assessment of how well the model represents the structural properties of the observed network. Therefore, new networks are generated from the fitted model, and structural statistics of the observed network are compared to the statistics calculated based on the generated networks [49]. Here, we check the statistics *degree*, i.e the distribution of degrees of nodes in a network. For this, we use the R package *statnet *facilitating the simulation of networks from the ERGMs with MCMC [50].

## Results and Discussion

The Bayesian inference of the p2-model for an undirected network was applied to the human protein interaction network (see Data). The code for the software OpenBUGS is available on [51]. We used 50000 MCMC iterations for the 'burn-in' and 100000 iterations for the estimation of parameters. ('Burn-in' period of the MCMC are the first iterations which are used for the chain to get to convergence sufficiently away from its initial values, but are discarded in the estimations of the posterior values). The classification of proteins into ordered and disordered based on their sequences was performed with the classifier POODLE-W (see Methods). We obtained 130 proteins (out of total 401) predicted to be disordered (see Additional Files 2 and 3).

The mean (standard deviation) of the density parameter *θ*, estimated from the Markov chain, is -5.68 (0.13). This means that, by absence of the sociality parameters *α*, the probability *p *of each edge in the network is 0.0034 (calculated by *log*() = -5.68). This probability can produce only 287 edges out of  = 80200 possible. The probability of edges further increases by positive effect of the nodes with high sociality. There were 177 nodes (from total 401) found with positive estimated means of sociality parameter *α*, whereas 69 of them had 2.5% quantile greater than 0. We propose to consider these 69 nodes as having the true positive influence on the formation of edges in the protein interaction network and call the respective proteins *social*. The identifiers of the proteins with the estimated means of their sociality parameters sorted in the decreasing order, as well as the estimated standard deviations, 2.5% and 97.5% quantiles, the proteins' degrees of connectivity in the network and the disorder class labels are demonstrated in Table 1.

**Table 1:** Social proteins in the human protein interaction network.

Protein	Symbol	Degree	alpha mean	sd	2.5%quantile	97.5%quantile	Disorder
NP_473376.1	UNC119	56	3.48	0.13	3.14	3.70	1

CAD39125.1	RIF1	53	3.41	0.13	3.06	3.63	1

AAH12509.1	EEF1A1	49	3.30	0.14	3.03	3.57	0

AAH13918.1	EEF1G	41	3.06	0.14	2.77	3.34	0

NP_000537.2	TP53	36	2.89	0.15	2.51	3.13	1

AAK55500.1	CRMP1	36	2.89	0.15	2.59	3.18	0

BAA92615.1	KIAA1377	35	2.85	0.15	2.47	3.10	1

NP_002816.1	PTN	34	2.81	0.15	2.42	3.06	1

NP_036564.1	SETDB1	32	2.73	0.16	2.34	2.99	1

XP_351098.1	CHD3	30	2.65	0.16	2.25	2.91	1

NP_005068.2	TLE1	29	2.61	0.16	2.21	2.87	1

NP_057144.1	CGI125	28	2.56	0.17	2.23	2.88	0

NP_009107.1	C14orf1	26	2.47	0.17	2.13	2.80	0

NP_874368.1	HTATIP	25	2.42	0.17	2.08	2.74	0

NP_002961.1	SAT	23	2.31	0.18	1.96	2.65	0

NP_003371.1	VIM	22	2.26	0.18	1.82	2.56	1

NP_004146.1	SERPINB9	20	2.14	0.19	1.76	2.49	0

AAH33561.1	DKFZP564O0523	19	2.08	0.19	1.69	2.44	0

NP_006824.2	COPS6	18	2.01	0.20	1.62	2.39	0

O00231	PSMD11	18	2.01	0.19	1.62	2.38	0

NP_009153.2	ZHX1	17	1.95	0.20	1.48	2.28	1

NP_002564.1	PAFAH1B3	17	1.94	0.20	1.55	2.32	0

NP_008896.1	ZNF24	16	1.87	0.20	1.39	2.22	1

NP_001060.1	TUBB	15	1.79	0.21	1.37	2.19	0

NP_000994.1	RPLP1	14	1.72	0.21	1.21	2.08	1

NP_006640.2	SDCCAG16	13	1.63	0.22	1.11	2.00	1

NP_777576.1	UBR1	13	1.63	0.22	1.18	2.04	0

P04183	TK1	13	1.63	0.22	1.17	2.05	0

NP_002937.1	RPA2	13	1.62	0.22	1.18	2.05	0

NP_006312.1	ARIH2	12	1.53	0.23	1.07	1.97	0

NP_002281.1	LAMA4	12	1.53	0.23	1.06	1.96	0

NP_060267.2	BTBD2	11	1.44	0.24	0.96	1.88	0

NP_003940.2	HAP1	11	1.43	0.24	0.88	1.83	1

Q9Y2X7	GIT1	11	1.43	0.23	0.96	1.88	0

NP_002037.2	GAPD	11	1.43	0.24	0.95	1.88	0

NP_004630.2	BAT3	10	1.33	0.25	0.75	1.74	1

Q13332	PTPRS	10	1.32	0.24	0.83	1.78	0

NP_005878.1	DLEU1	10	1.32	0.25	0.81	1.79	0

BAB14293.1	ASC1p100	10	1.32	0.24	0.82	1.79	0

NP_001316.1	CSTF2	9	1.21	0.26	0.62	1.65	1

NP_002148.1	HSPE1	9	1.21	0.25	0.68	1.69	0

NP_061730.1	PCDHA4	9	1.20	0.26	0.68	1.69	0

CAD97612.1	IMMT	8	1.08	0.27	0.45	1.54	1

NP_057103.1	LUC7L2	8	1.08	0.27	0.45	1.53	1

CAB72445.1	BRD7	8	1.08	0.27	0.45	1.53	1

NP_061960.1	ARFRP2	8	1.08	0.27	0.53	1.58	0

NP_002203.1	ITGB4BP	8	1.07	0.27	0.52	1.57	0

NP_005997.2	ZNF145	8	1.07	0.27	0.52	1.58	0

NP_008998.1	MYST2	7	0.93	0.29	0.28	1.42	1

NP_060719.3	CDK5RAP2	7	0.93	0.29	0.28	1.41	1

AAB96331.1	APLP1	7	0.93	0.29	0.27	1.41	1

NP_001253.1	CDKN2C	7	0.93	0.29	0.34	1.46	0

NP_036569.1	SNAPAP	7	0.93	0.28	0.34	1.46	0

NP_000362.1	TTR	7	0.93	0.29	0.34	1.46	0

Q96RU7	C20orf97	7	0.93	0.28	0.34	1.46	0

AAH33094.1	IKBKAP	7	0.93	0.29	0.34	1.46	0

NP_004441.1	ERH	7	0.93	0.29	0.33	1.46	0

NP_005774.2	APACD	7	0.93	0.28	0.35	1.45	0

NP_001782.1	CDC42	7	0.93	0.29	0.33	1.46	0

NP_002613.2	PFDN1	6	0.76	0.31	0.07	1.28	1

NP_004302.1	ARL3	6	0.76	0.30	0.14	1.33	0

NP_002680.2	POLA2	6	0.76	0.30	0.13	1.32	0

NP_001460.1	G22P1	6	0.76	0.30	0.13	1.33	0

NP_444252.1	PFN2	6	0.76	0.30	0.13	1.33	0

NP_006077.1	TUBB4	6	0.76	0.31	0.13	1.33	0

NP_005251.1	GDF9	6	0.76	0.30	0.13	1.32	0

NP_002936.1	RPA1	6	0.76	0.30	0.13	1.32	0

NP_071921.1	FTS	6	0.76	0.30	0.13	1.32	0

AAH08720.1	CRELD1	6	0.76	0.30	0.13	1.31	0

Table 1 contains the list of 69 social proteins revealed by the statistical analysis. NCBI identifiers (RefSeqIds) and symbols (as used by [36]) of the proteins are displayed. For each protein, the degree of the respective node in the network is showed. Columns 4-7 present the estimated mean, standard deviation, 2.5% and 97.5% quantiles of the sociality parameter *α*. The last column contains the disorder group of each protein. (The proteins are sorted in the decreasing order of the mean of the sociality parameter.)

The degrees of connectivity of the social proteins are greater or equal 6. The definition of hubs as the top 20% of high-degree proteins would suggest 80 proteins, hence also with degree 5.

From the 69 social proteins, 23 are disordered. The corresponding functional keywords obtained from Swiss-Prot [52] are presented in the Additional File 4. It can be seen, that these proteins are involved in biological processes like transcription, transcription regulation, cell cycle, mRNA processing, ribosome biogenesis, Wnt signaling. The proteins possess coiled coil, Zinc-finger, repeat domains, as well as homeobox, EGF-like and bromodomain. The majority of proteins are associated with alternative splicing and polymorphism i.e. coding sequence diversity that potentially relies on protein intrinsic disorder. The fact that alternatively splicing events map to regions of disorder much more often than to regions of structure was emphasized in [53]. The combination of disorder and alternative splicing was proposed to provide a mechanism for generating signaling diversity and enabling the evolution of cell differentiation and multicellularity. Alternative splicing and polymorphism are proposed to increase proteome size and regulatory and signaling network complexity, thus generating organism complexity.

We used the p2-model in order to incorporate in it exogeneous nodal attributes, namely the disorder groups of the respective proteins. The parameter *γ *reflecting the effect of the disorder on the sociality of the nodes in the network was estimated 0.06 (0.095), thus revealing that protein disorder has the positive effect on the nodal sociality, and hence on the probability of edges in the network. However, the 2.5% quantile of *γ *was estimated as -0.105 and the 97.5% quantile was 0.275. This indicates that the effect of the disorder is still small and cannot fully explain the sociality of nodes in the network. The parameters a, a kind of 'residuals' after considering the effect of disorder, appeared to have high estimated values. The present finding supports the hypotheses that proteins folding property disorder plays a role in the protein network achitecture, however, other reasons enabling proteins to interact with large numbers of proteins should also be considered.

The goodness-of-fit plot for the degree statistics, which compares the degree distributions of the observed and simulated networks, is presented in the Additional File 5. There were 100 networks generated from the model. It can be seen, that the model slightly under-predicts the degrees of the nodes, and hence, the number of edges in the network.

**Figure 1:**
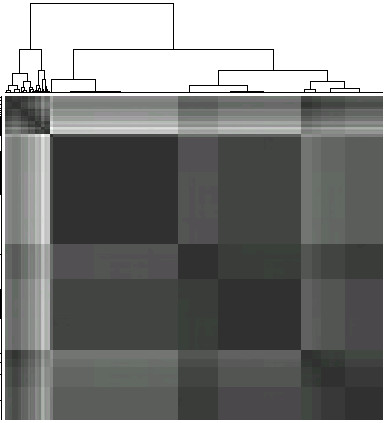
**Heatmap and cluster diagram demonstrating the clustering of proteins based on their sociality parameters**. Figure 1 presents the heatmap and the cluster diagram depicting groups of structurally similar proteins in the protein interaction network obtained by clustering the sociality parameters of the proteins.

Further on, we calculated the Euclidian distance matrix between the sociality parameters of all the nodes in the network, and used it to cluster the nodes by hierarchical complete-linkage clustering. The heatmap with the cluster diagram on top is presented in Figure 1. The clustering suggests five groups of nodes, whereby the nodes in each group possess similar patterns of connetivity. Table 2 lists the clusters, their sizes and the range of degrees of the nodes contained in each cluster, respectively. Moreover, to investigate the common functional properties of proteins contained in each cluster, we performed the gene ontology analysis of the respective genes. Namely, we obtained the GO terms significantly enriched in each cluster as compared to other proteins in the set (we employed the R package GOSim [54]). The taxonomy *molecular function *("MF") was used. The enriched GO terms for each cluster are displayed in the last column of Table 2. The inspection of the GO terms suggests that "very high"-connected proteins (Cluster 1) are involved in remodeling of chromatin structure, modifications of histones, translation elongation, DNA methylation, DNA replication, alternative splicing, thus related to transcriptional machinery i.e. to fundamental genetic processes. The proteins are associated with development, differentiation, response to diverse cellular stresses etc. Cluster 2 ("high"-degree proteins) probably contains proteins with a broad diversity of functions - only one enriched GO term "zinc ion binding" was found. Cluster 3 with "moderate"-degree proteins is associated with signal transduction and, via hormone binding, with metabolic processes. Clusters 4 and 5 ("low"- and "very low"-connected proteins) are involved in the specific regulation of transcription. The proteins exhibit transcription factor activity. One additional function is the structural molecule activity i.e. contribution to the structural integrity of a complex or assembly.

**Table 2:** Clusters of structurally similar proteins.

Cluster	Size	Degrees	GO Terms enriched (Molecular Function, "MF")
Cluster 1	16	22-56	GO:0000739 DNA strand annealing activity
			GO:0003682 chromatin binding
			GO:0003746 translation elongation factor activity
			GO:0004145 diamine N-acetyltransferase activity
			GO:0004157 dihydropyrimidinase
			GO:0004864 protein phosphatase inhibitor activity
			GO:0016455 RNA polymerase II transcription mediator activity
			GO:0018024 histone-lysine N-methyltransferase activity
			GO:0050681 androgen receptor binding

Cluster 2	32	8-20	GO:0008270 zinc ion binding

Cluster 3	86	4-7	GO:0003899 DNA-directed RNA polymerase activity
			GO:0005125 cytokine activity
			GO:0019900 kinase binding
			GO:0042562 hormone binding

Cluster 4	131	2-3	GO:0003777 microtubule motor activity
			GO:0005088 Ras guanyl-nucleotide exchange factor activity

Cluster 5	136	1	GO:0003700 transcription factor activity
			GO:0003723 RNA binding
			GO:0003779 actin binding
			GO:0005198 structural molecule activity

Table 2 presents the groups of proteins obtained by clustering the respective sociality parameters. The number of proteins in each cluster, their degrees and molecular functions found by GO-significance analysis are displayed.

**Figure 2:**
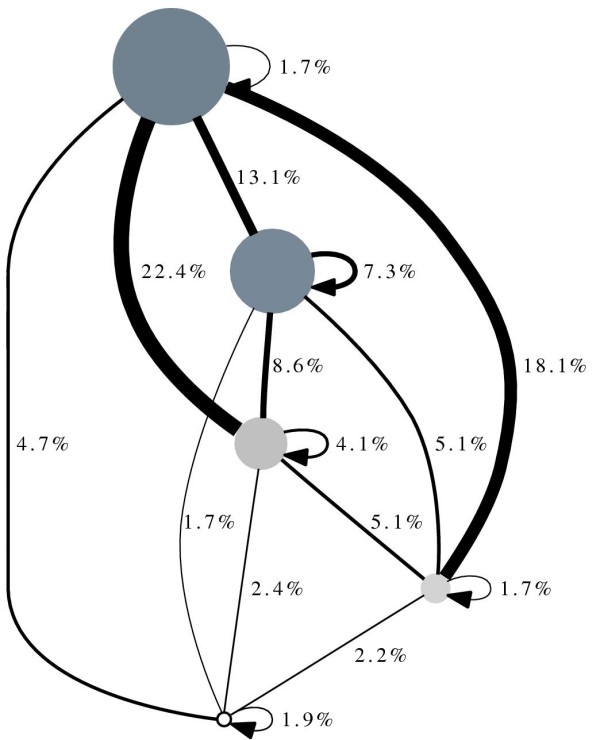
**Network diagram**. Figure 2 presents the diagram summarizing the protein interaction network as five groups of structurally similar proteins with interaction flows between them. The size and grey color intensity of the nodes reflect the degrees of proteins contained in each group. The width and labeling of the ties reflect the number of interactions between proteins belonging to two groups (demonstrated as percentage of the total network interactions). The arrows depict the self-interaction of each group.

We summarized the whole protein network as a diagram representing clusters (i.e. groups of structurally similar proteins) and relations between them (Figure 2). The size and grey color intensity of the nodes in the diagram reflect the degrees of the network nodes contained in each cluster. The width of the ties in the diagram reflects the number of network interactions between the nodes belonging to two clusters (presented as percentage of the total 911 interactions). Interactions between nodes of the same cluster are displayed with the arrows. All protein interactions are now distributed as flows in the diagram. The diagram reveals the global organization of the network, abstracted from the level of individual nodes. The flows indicate that Cluster 1 is tightly connected to all other clusters, thus playing a role important to all proteins in the network. Indeed, it is associated with the basal, generally important process - transcriptional control. Interestingly, Cluster 2 is much higher connected to itself than other clusters, depicting the presence of a tight middle-layer of proteins in the network. Longer path "moderate"-"very high"-"low"-degree proteins might indicate a certain sequence of processes in the proteins interactivity. It can be postulated that the connectivity of proteins is related to the generality (or specificity) of their functions.

Although we used a subpart of all available protein interactions, the most reliable one, we believe that the tendencies revealed here will remain valid, as the biological knowledge will accumulate. Our method can be readily applied to larger networks.

## Conclusions

We proposed a method for the analysis of biological networks based on the statistical modelling with the exponential random graph model, namely p2-model. We applied the method to a human protein interaction network. Bayesian approach enabled flexible model definitions and facilitated the model inference.

The statistical modelling handles the observed network as a probabilistic outcome of an underlying process of network formation. It allows to make explicit assumptions on the model that generated the network data and precise statements on the validity of hypotheses about the network.

In contrast to the descriptive methods of the analysis of biological networks, the ERGM approach attempts to address the origin of global network architecture and to explain the emergence of the network from local interaction patterns. It is more biologically plausible, that biological phenomena are reflected in the local network structural configurations, than that some proteins are responsible for the organization of the global network structure. It is a good challenge to reveal, what biological phenomena or aspects of cellular life are essential from the local structural point of view.

Previously, the problem of network formation was regarded by the evolutionary approaches. The *duplication-divergence *model considered gene duplications and gain and loss of protein interactions through mutations as the driving force shaping the structure of the network ([55,56]). In [57], the domain-based evolutionary model was introduced. The goal was mainly to explain (or reproduce) the scale-free property of the protein network.

The exponential random graph model tries to describe the structural properties of the network with a limited number of parameters. Here, we used one global parameter representing the density of interactions in a network, and local, node-specific parameters reflecting the propensity of each individual node to connect to other nodes. i.e. the model is node-oriented. When positive, the node-specific parameters indicate that the respective nodes contribute to the formation of edges in the network. This feature suggests a novel definition of essential nodes in the network, namely social, as revealed based on the parameters of the fitted model. Previously, hubs, as highly interactive nodes occupying central positions in the network, were considered to be essential as they are important for the maintenance of the global network architecture - the scale-free architecture. Removing hubs increases the proportion of unreachable nodes and the shortest path lengthes in the network more than removing non-hubs [58]. The previous definitions of essential nodes in biological networks are based on descriptive measures, in contrast to the generic approach proposed here.

The main advantage of our approach is that it provides a possibility for biological hypothesis testing. Attributes of nodes (as well as of edges) can be inserted into the model to explore the role of these properties in the emergence of the network. Here, we examined the influence of protein disorder on the nodal connectivity and revealed the positive effect of the disorder. We confirmed the recent idea that protein disorder provides a mechanism facilitating proteins binding diversity [33]. However, protein disorder exhibits an effect, but do not fully explains the proteins connectivity, henceforth other information on proteins should be examined in the context of the protein interaction network.

Notably, previous approaches were based on the paradigm "from the structural importance of nodes to the functional". Our work challenges an opposed way of thinking, and enables to deduce, which properties of biological entities are important for the structure of the biological network.

In [59], the role of intrinsic disorder was studied with respect to the "date" and "party" classifications of proteins. Date proteins are those building transient interactions with their partners, while party proteins interact with their partners simultaneously (this classification of proteins was done based on the correlation of mRNA expression). It was suggested that intrinsic disorder in date proteins may facilitate transient interactions. The date and party classification of human proteins can be incorporated in our framework together with the disorder information, to explore the role of both properties in the organization of the protein interaction network.

The results of [60] indicate that multi-domain proteins are overrepresented among hubs in the interaction network of yeast. Also, repeated structural motifs are enriched in hub proteins. E.g. the WD40-repeat protein acts in the formation of a multi-protein corepressor complex, where the repeating units serve as a rigid scaffold for protein interactions. The information about domain content and repeats would be valuable to consider in our modelling.

Whereas previous works firstly determined hubs by setting an arbitrary cutoff and then related particular biological properties to them (e.g. "date" and "party" hubs, singlish- and multi-interface hubs, see [59,61,62]), our approach allows each protein in the network to exhibit an impact according to its biological feature.

Further examples of biological properties, which could be incorporated in our model, are genes biological essentiality, as well as proteins functional groups. The relation between two proteins is actually explained by a biological function they are involved in (regulatory, signalling, metabolic etc.). Inference of this function remains a great challenge. The proposed statistical modelling approach allows to check, what qualities of proteins stimulate the interactions between them, what functional characteristics determine the emergence and the structure of the protein network.

We provided a novel way of revealing the organizational principles of a biological network as being constructed from a few building blocks comprizing individuals with equivalent connectivity.

The ERGM approach provides a link between the *network motifs *research emergent in the systems biology and the generic perspective. Various types of network motifs significantly enriched as compared to randomized networks were revealed by the study of transcription regulatory network of *E.coli*: feed-forward loops (FFL), single-input modules (SIM), dense overlapping regulons (DOR) [63]. The ERGM modelling of networks offers a natural way of assessing importance of the network motifs. Moreover, it should explain the biological reasons for the formation of motifs. Using the terms of the statistical network analysis field, the feed-forward loop is the triangle, the SIM is the out-star. The work of [17] was a first attempt to learn the higher-order exponential random graph models from biological networks and to obtain the importance of these structural configurations. However, only basic configurations (edges and 2-pathes) showed a positive propensity to be present in the networks. This suggests that further configurations and their respective statistics, characteristic to the biological networks, must be developed (e.g. longer paths). Novel, four-element motifs Bi-fan, Bi-FFL and Bi-parallel were found recently in the combined transcription factor-binding and phosphorylation network of yeast [64].

To summarize, the statistical modelling of networks presented here provides a framework combining three research directions: 1) the generic analysis of networks, 2) the structural analysis of networks and 3) the biological analysis of individual entities, all aiming at understanding the functional principles of complex biological systems.

The approach described here is very promising for the *comparative analysis *of networks involving the same nodes, since it provides the parameters on which the networks can be compared. An important application will be the *cross-species *comparative analysis of biological networks (proteins interaction, metabolic etc.) involving conserved nodes. Also, the approach provides new perspectives on the *dynamic network analysis *that investigates how network structure changes over time.

## Authors' contributions

SB developed the concept of the statistical analysis of biological networks, elaborated the Bayesian approach, performed the analysis, interpreted the results and drafted the manuscript. AB carried out the prediction of proteins disorder and participated in drafting the manuscript. RE have given approval of the manuscript. All authors read and improved the final manuscript.

## Supplementary Material

Additional file 1 The file contains the NCBI identifiers (RefSeqIds) and symbols of 401 proteins of the human interaction network. Click here for file

Additional file 2 The file contains output from the tool POODLE-W in FASTA format. The predicted probability of each protein to be mostly disordered is presented as e.g. DR_PROB: 0.996. Click here for file

Additional file 3 The file contains the NCBI identifiers (RefSeqIds) of 130 proteins classified to be disordered. Click here for file

Additional file 4 The file contains Swiss-Prot functional keywords of 23 disordered proteins from 69 social proteins revealed by our approach. Click here for file

Additional file 5 The file represents the goodness-of-fit plot which compares degree distribution between observed protein interaction network and 100 networks simulated from the fitted model. X-axis represents the degree values, Y-axis represents the fraction of nodes exhibiting that value. Solid line represents original network; boxplots display the distribution of the degree statistics across the 100 simulated networks. Click here for file

## References

[B1] Barabasi AL Albert R Emergence of scaling in random networks Science 1999 286 5439 509 512 10.1126/science.286.5439.509 10521342

[B2] Barabasi AL Oltvai ZN Network biology: understanding the cell's functional organization Nat Rev Genet 2004 5 101 113 10.1038/nrg1272 14735121

[B3] Jeong H Mason S Barabasi A Oltvai Z Lethality and centrality in protein networks Nature 2001 411 6833 41 42 10.1038/35075138 11333967

[B4] Singh GP Dash D Intrinsic disorder in yeast transcriptional regulatory network Proteins 2007 15; 68 3 602 605 10.1002/prot.21497 17510967

[B5] Haynes C Oldfield CJ Ji F Klitgord N Cusick ME Radivojac P Uversky VN Vidal M Iakoucheva LM Intrinsic disorder is a common feature of hub proteins from four eukaryotic interactomes PLoS Comput Biol 2006 2 8 890 901 10.1371/journal.pcbi.0020100 PMC1526461 16884331

[B6] Girvan M Newman ME Community structure in social and biological networks Proc Natl Acad Sci USA 2002 99 12 7821 7826 10.1073/pnas.122653799 12060727 PMC122977

[B7] Yu H Kim PM Sprecher E Trifonov V Gerstein M The importance of bottlenecks in protein networks: correlation with gene essentiality and expression dynamics PLoS Comput Biol 2007 20 3(4):e59 713 720 10.1371/journal.pcbi.0030059 PMC1853125 17447836

[B8] Wuchty S Stadler PF Centers of complex networks J Theor Biol 2003 7; 223 1 45 53 10.1016/S0022-5193(03)00071-7 12782116

[B9] Junker BH Koschützki D Schreiber F Exploration of biological network centralities with CentiBiN BMC Bioin-formatics 2006 21 7 219 10.1186/1471-2105-7-219 PMC1524990 16630347

[B10] Potapov A Goemann B Wingender E The pairwise disconnectivity index as a new metric for the topological analysis of regulatory networks BMC Bioinformatics 2008 9 227 10.1186/1471-2105-9-227 18454847 PMC2396639

[B11] Wasserman S Faust K Social network analysis: methods and applications 1999 Cambridge Univ Press

[B12] Wasserman S Robins G J CP An introduction to random graphs, and p*. In Models and Methods in Social Network Analysis 2005 Cambridge Univ Press

[B13] Holland PW Leinhardt S An exponential family of probability distributions for directed graphs Journal of the American Statistical Association 1981 76 33 50 10.2307/2287037

[B14] Frank O Strauss D Markov graphs Journal of the American Statistical Association 1986 81 832 842 10.2307/2289017

[B15] Snijders TAB Pattison PE Robins GL Handcock MS New Specifications for Exponential Random Graph Models Sociological Methodology 2004 99 153

[B16] Robins G Snijders T Wang Handcock MS Pattison P Recent developments in exponential random graph (p*) models for social networks Social Networks 2006 29 192 215 10.1016/j.socnet.2006.08.003

[B17] Saul Z Filkov V Exploring biological network structure using exponential random graph models Bioinformatics 2007 23 19 2604 2611 10.1093/bioinformatics/btm370 17644557

[B18] van Duijn M Snijders T Zijlstra B p2: a random effects model with covariates for directed graphs Statistica Neerlandica 2004 58 234 254 10.1046/j.0039-0402.2003.00258.x

[B19] Wright P Dyson H Intrinsically unstructured proteins: re-assessing the protein structure-function paradigm J Mol Biol 1999 22; 293 2 321 31 10.1006/jmbi.1999.3110 10550212

[B20] Uversky V Gillespie J Fink A Why are "natively unfolded" proteins unstructured under physiologic conditions? Proteins 2000 15; 41 3 415 27 10.1002/1097-0134(20001115)41:3<415::AID-PROT130>3.0.CO;2-7 11025552

[B21] Uversky V What does it mean to be natively unfolded? Eur J Biochem 2002 269 1 2 12 10.1046/j.0014-2956.2001.02649.x 11784292

[B22] Dyson H Wright P Intrinsically unstructured proteins and their functions Nat Rev Mol Cell Biol 2005 6 3 197 208 10.1038/nrm1589 15738986

[B23] Oldfield CJ Cheng Y Cortese MS Brown CJ Uversky VN Dunker AK Comparing and combining predictors of mostly disordered proteins Biochemistry 2005 15; 44 6 1989 2000 10.1021/bi047993o 15697224

[B24] Dunker A Obradovic Z Romero P Garner EC Brown CJ Intrinsic protein disorder in complete genomes Genome Inf Ser 2000 11 161 171 11700597

[B25] Iakoucheva LM Brown CJ Lawson JD Obradovic Z Dunker AK Intrinsic disorder in cell-signaling and cancer-associated proteins J Mol Biol 2002 323 573 584 10.1016/S0022-2836(02)00969-5 12381310

[B26] Cheng Y LeGall T Oldfield C Dunker A Uversky V Abundance of intrinsic disorder in protein associated with cardiovascular disease Biochemistry 2006 45 35 10448 60 10.1021/bi060981d 16939197

[B27] Dunker AK Brown CJ Lawson J Iakoucheva LM Obradovic Z Intrinsic disorder and protein function Biochemistry 2002 41 21 6573 6582 10.1021/bi012159+ 12022860

[B28] Xie H Vucetic S Iakoucheva LM Oldfield CJ Dunker AK Uversky VN Obradovic Z Functional anthology of intrinsic disorder 1. Biological processes and functions of proteins with long disordered regions J Proteome Res 2007 6 5 1882 98 10.1021/pr060392u 17391014 PMC2543138

[B29] Vucetic S Xie H Iakoucheva LM Oldfield CJ Dunker AK Obradovic Z Uversky VN Functional anthology of intrinsic disorder 2. Cellular components, domains, technical terms, developmental processes, and coding sequence diversities correlated with long disordered regions J Proteome Res 2007 6 5 1899 916 10.1021/pr060393m 17391015 PMC2588346

[B30] Xie H Vucetic S Iakoucheva LM Oldfield CJ Dunker AK Obradovic Z Uversky VN Functional anthology of intrinsic disorder 3. Ligands, post-translational modifications, and diseases associated with intrinsically disordered proteins J Proteome Res 2007 6 5 1917 32 10.1021/pr060394e 17391016 PMC2588348

[B31] Dunker A Obradovic Z The protein trinity-linking function and disorder Nat Biotechnol 2001 19 9 805 6 10.1038/nbt0901-805 11533628

[B32] Radivojac L Pand Iakoucheva Oldfield C Obradovic Z Uversky V AK D Intrinsic disorder and functional pro-teomics Biophys J 2007 1; 92 5 1439 56 10.1529/biophysj.106.094045 PMC1796814 17158572

[B33] Dunker AK Cortese M Romero P Iakoucheva L Uversky V Flexible nets The roles of intrinsic disorder in protein interaction networks FEBS J 2005 272 20 5129 5148 10.1111/j.1742-4658.2005.04948.x 16218947

[B34] Dosztanyi Z Chen J Dunker AK Simon I Tompa P Disorder and sequence repeats in hub proteins and their implications for network evolution J Proteome Res 2006 5 11 2985 2995 10.1021/pr060171o 17081050

[B35] Patil A Nakamura H Disordered domains and high surface charge confer hubs with the ability to interact with multiple proteins in interaction networks FEBS Lett 2006 580 8 2041 2045 10.1016/j.febslet.2006.03.003 16542654

[B36] Stelzl U A human protein-protein interaction network:a resource for annotating the proteome Cell 2005 23; 122 6 830 832 10.1016/j.cell.2005.08.029 16169070

[B37] NCBI http://www.ncbi.nlm.nih.gov/entrez/

[B38] POODLE http://mbs.cbrc.jp/poodle/poodle-l.html

[B39] Shimizu K Muraoka Y Hirose S Noguchi T Feature Selection Based on Physicochemical Properties of Redefined N-term Region and C-term Regions for Predicting Disorder Proc of IEEE CIBCB 2005 262 267

[B40] Hirose S Shimizu K Kanai S Kuroda Y Noguchi T POODLE-L: a two-level SVM preidction system for reliably predicting long disordered regions Bioinformatics 2007 23 16 2046 53 10.1093/bioinformatics/btm302 17545177

[B41] Shimizu K Muraoka Y Hirose S Tomii K Noguchi T Predicting mostly disordered proteins by using structure-unknown protein data BMC Bioinformatics 2007 8 78 10.1186/1471-2105-8-78 17338828 PMC1838436

[B42] Wasserman S Pattison P Logit models and logistic regressions for social networks Psychometrika 1996 61 3 401 425 10.1007/BF02294547 10613111

[B43] Strauss D Ikeda M Pseudolikelihood estimation for social networks Journal of the American Statistical Association 1990 85 204 212 10.2307/2289546

[B44] Snijders TAB Markov Chain Monte Carlo estimation of exponential random graph models Journal of Social Structure 2002 3 2

[B45] Gill PS Swartz TB Bayesian analysis of directed graphs data with applications to social networks Journal of the Royal Statistical Society: Series C (Applied Statistics) 2004 53 2 249 260 10.1046/j.1467-9876.2003.05215.x

[B46] Fienberg SE Wasserman S Leinhardt S Categorical data analysis of single sociometric relations Sociological Methodology 1981 12 San Francisco: Jossey-Bass 156 192 10.2307/270741

[B47] Thomas A O Hara B Ligges U Sturtz S Making BUGS Open R News 2006 6 12 17

[B48] CODA http://cran.r-project.org

[B49] Hunter DR Goodreau SM Handcock MS Goodness of fit of social network models Journal of the American Statistical Association 2008 103 481 248 258 10.1198/016214507000000446

[B50] Handcock MS Hunter DR Butts CT Goodreau SM M M statnet: Software tools for the statistical modeling of network data 2003 http://www.ncbi.nlm.nih.gov/entrez/10.18637/jss.v024.i01 PMC2447931 18618019

[B51] Author's homepage http://www.dkfz.de/tbi/people/homepages/bulashev/Supplementary

[B52] Swiss-Prot http://www.ebi.ac.uk/swissprot/

[B53] Romero P Zaidi S Fang Y Uversky V Radivojac P Oldfield C Cortese M Sickmeier M LeGall T Obradovic Z Alternative splicing in concert with protein intrinsic disorder enables increased functional diversity in multicellular organisms Proc Natl Acad Sci USA 2006 103 22 8390 5 10.1073/pnas.0507916103 16717195 PMC1482503

[B54] Fröhlich H Speer N Poustka A Beissbarth T GOSim-an R-package for computation of information theoretic GO similarities between terms and gene products BMC Bioinformatics 2007 8 166 10.1186/1471-2105-8-166 17519018 PMC1892785

[B55] Berg J Laessig M Wagner A Structure and evolution of protein interaction networks: a statistical model for link dynamics and gene duplications BMC Evolutionary Biology 2004 4 1 51 10.1186/1471-2148-4-51 15566577 PMC544576

[B56] Ispolatov I Krapivsky P Yuryev A Duplication-divergence model of protein interaction network Phys Rev 2005 E71 10.1103/PhysRevE.71.061911 PMC2092385 16089769

[B57] Nacher J Hayashida M Akutsu T Topological aspects of protein networks Studies in Computational Intelligence 2007 56 147 158 full_text

[B58] Albert R Jeong H Barabasi AL Error and attack tolerance of complex networks Nature 2000 406 378 382 10.1038/35019019 10935628

[B59] Singh G Ganapathi M Dash D Role of intrinsic disorder in transient interactions of hub proteins Proteins 2006 1; 66 4 761 765 10.1002/prot.21281 17154416

[B60] Ekman D Light S Björklund A Elofsson A What properties characterize the hub proteins of the protein-protein interaction network of Saccharomyces cerevisiae? Genome biology 2006 7 6 R45 10.1186/gb-2006-7-6-r45 16780599 PMC1779539

[B61] Kim P Lu L Xia Y Gerstein M Relating three-dimensional structures to protein networks provides evolutionary insights Science 2006 314 5807 1938 41 10.1126/science.1136174 17185604

[B62] Kim P Sboner A Xia Y Gerstein M The role oif disorder in interaction networks: a structural analysis Molecular Systems Biology 2008 4 179 10.1038/msb.2008.16 18364713 PMC2290937

[B63] Shen-Orr SS Milo R Mangan S Alon U Network motifs in the transcriptional regulation network of *Escherichia coli* Nat Genet 2002 31 1 64 68 10.1038/ng881 11967538

[B64] Zhu X Gerstein M Snyder M Getting connected: analysis and principles of biological networks, Review Genes and Development 2007 21 1010 1024 10.1101/gad.1528707 17473168

