# Supplementary figures and images for: Bayesian statistical modelling of human protein interaction network incorporating protein disorder information
Source: BMC Bioinformatics. 2010 Jan 25;11:46. doi: 10.1186/1471-2105-11-46 (PMC2831004; doi:10.1186/1471-2105-11-46)

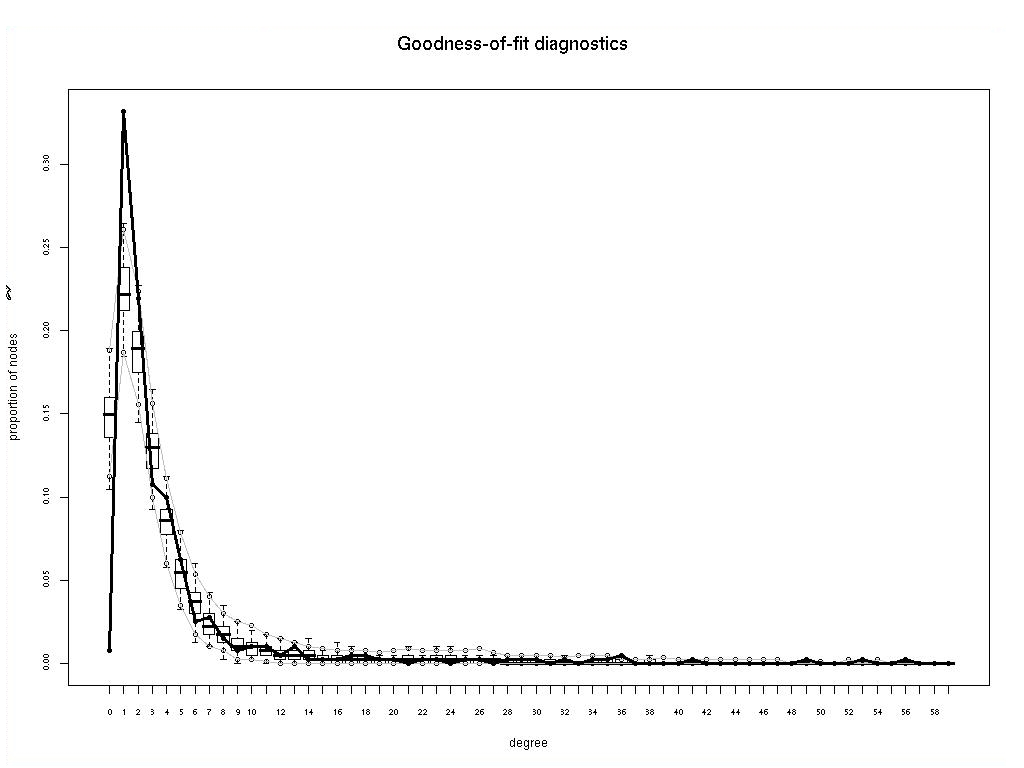

Supplement: Additional file 5 — The file represents the goodness-of-fit plot which compares degree distribution between observed protein interaction network and 100 networks simulated from the fitted model. X-axis represents the degree values, Y-axis represents the fraction of nodes exhibiting that value. Solid line represents original network; boxplots display the distribution of the degree statistics across the 100 simulated networks. [file 1471-2105-11-46-S5.JPEG]
